# Association of genetic polymorphisms of SelS with Type 2 diabetes in a Chinese population

**DOI:** 10.1042/BSR20181696

**Published:** 2018-11-28

**Authors:** Long Zhao, Ying-Ying Zheng, You Chen, Yi-Tong Ma, Yi-Ning Yang, Xiao-Mei Li, Xiang Ma, Xiang Xie

**Affiliations:** 1 Department of Coronary Artery Disease, Heart Center, First Clinical Medical College and First Affiliated Hospital of Xinjiang Medical University, Urumqi 830011, P.R. China; 2 Department of Cardiology, First Affiliated Hospital of Zhengzhou University, Zhengzhou 450002, P.R. China

**Keywords:** Genetic polymorphisms, Sels, Type 2 diabetes

## Abstract

**Background:** Selenoprotein S (SelS) gene expression is positively correlated to triglyceride (TG) concentrations and is associated with diabetes in animal model. However, the relationship between genetic polymorphisms of SelS and Type 2 diabetes (T2DM) remains unclear. **Methods:** In the present study, we genotyped four single nucleotide polymorphisms (rs12910524, rs1384565, rs2101171, rs4965814) of *SelS* gene using TaqMan genotyping method in a case–control study (1947 T2DM patients and 1639 control subjects). **Results:** We found both rs1384565 CC genotype (12.1 compared with 6.6%, *P*<0.001) and C allele (35.2 compared with 24.4%, *P*<0.001) were more frequent in the T2DM patients than in the controls. Logistic regression analysis suggested after adjustment of other confounders, the difference remained significant between the two groups (CC compared with TT, *P*=0.002, OR = 1.884, 95% CI: 1.263–2.811; CT compared with TT, *P*<0.001, OR = 1.764, 95% CI: 1.412–2.204). **Conclusion:** The present study suggested that genetic polymorphisms of SelS were associated with T2DM in a Chinese population.

## Introduction

Type 2 diabetes (T2DM) is a multifactor complex disease resulting from the interaction between genetics and unhealthy lifestyle [1]. A large number of studies showed that many gene variations were associated with T2DM [2–6]. Jung et al. [7] performed a meta-analysis and found that middle-aged and elderly adults with the minor allele of ABCA1 rs2230806 have a lower risk of T2DM. Ding et al. [8] found rs7903146 in *TCF7L2* gene was significantly associated with T2DM in Caucasian, East Asian, South Asian, and other ethnicities. Sun et al. [9] found the polymorphism of rs266729 in adiponectin gene was associated with T2DM.

Selenoprotein S (SelS) is a transmembrane protein that is expressed in the liver, skeletal muscle, adipose tissue, pancreatic islets, kidney, and blood vessels [10,11]. Previous studies suggested that serum SelS level was associated with T2DM, dyslipidemia, and its macrovascular complications [12–14]. In a polygenic animal model of T2DM-Psammomys obesus, the SelS was found to be positively correlated to circulating triglyceride (TG) concentration [15]. Cox et al. [16] found genetic polymorphisms of SelS were associated with subclinical cardiovascular disease. Olsson et al. [17] suggested that *SelS* gene is expressed in subcutaneous adipose tissue and SelS genotypes are associated with metabolic risk factors. Our previous study also suggested genetic polymorphisms in SelS were associated with TG levels in healthy population [18]. Du et al. [19] suggested that SelS expression is increased in omental adipose tissue from diabetic subjects compared with that in nondiabetic controls. Also, SelS expression was found to be correlated with homeostasis model assessment of insulin resistance (HOMA-IR) [19]. Furthermore, Curran et al. [20] reported that a promoter polymorphism (rs28665122) in SelS was associated with inflammatory response, including elevated circulating levels of proinflammatory cytokines. These results suggested that SelS plays a role in inflammation and glycolipid metabolism. However, the relation between SelS genetic polymorphisms and T2DM remains unclear.

In the present study, we investigated the association between genetic polymorphisms of SelS and T2DM in a Chinese population.

## Methods

### Subjects

The study protocol was approved by the Ethics Committee of the First Affiliated Hospital of Xinjiang Medical University and was conducted according to the standards of the Declaration of Helsinki. Written informed consent was obtained from all the participants.

One thousand nine hundred and forty-seven Chinese patients diagnosed with T2DM at the First Affiliated Hospital of Xinjiang Medical University from January 2010 to December 2016 were recruited and acted as the case group. Subjects with T2DM were defined as those who had fasting plasma glucose (FPG) ≥ 7.0 mmol/l, or 2-h postload plasma glucose (2hPG) ≥ 11.1 mmol/l, or had hypoglycemic therapy history. One thousand six hundred and thirty-nine subjects with normal glucose tolerance (NGT) were designated the control group. NGT was defined as FPG < 6.1 mmol/l and 2hPG < 7.8 mmol/l.

### Genotyping

Blood samples were collected from all the participants, and genomic DNA was extracted from the peripheral blood leukocytes by DNA extraction Kit (Beijing Bioteke Co. Ltd., China). There are 190 SNPs for the human *SelS* gene listed in the National Center for Biotechnology Information SNP database (http://www.ncbi.nlm.nih.gov/ SNP). We also screened the data for the Tag SNPs on the International HapMap Project website (http://www.hapmap.org/). Using the Haploview 4.2 software and the HapMap phrase II database, we obtained four tagging SNPs (rs12910524, rs1384565, rs2101171, and rs4965814) for Chinese Han using minor allele frequency (MAF) ≥ 0.05 and linkage disequilibrium patterns with r^2^ ≥ 0.8 as a cutoff. Genotyping was confirmed using TaqMan® assays from Applied Biosystems following the manufacturer’s suggestions and analyzed in an ABI 7900HT Fast Real-Time PCR System as described previously [21].

### Biochemical analysis

Serum and plasma collected for measurement were immediately frozen at −80°C until detection. We measured the plasma concentration of total cholesterol (TC) and HDL cholesterol (HDL-C) and the serum concentration of creatinine and uric acid using equipment for chemical analysis (Dimension AR/AVL Clinical Chemistry System, Newark, NJ) employed by the Clinical Laboratory Department of the First Affiliated Hospital of Xinjiang Medical University.

### Statistical analyses

Analyses were carried out using SPSS version 17.0 (SPSS, Chicago, IL, U.S.A.). The Hardy–Weinberg equilibrium was assessed by chi-square analyses. Kolmogorov–Smirnov test was used to examine the normality of variables. Data were reported as mean plus or minus S.D. for normally distributed variables or as median (minimum to maximum) for non-normally distributed variables. Continuous variables were compared using Student’s *t*test, and non-normally distributed variables were analyzed by Mann–Whitney U test. Differences in enumeration data between T2DM patients and control subjects were analyzed using the chi-square test, as were differences in distributions of genotypes and alleles between T2DM patients and control subjects. Logistic regression analyses were used to assess the contribution of the major risk factors. Two-tailed *P*-values, 0.05, were considered significant.

## Results

### Characteristics of study participants

The clinical and metabolic characteristics of the studied population are shown in Table 1. There are significant differences between the T2DM group and the control group in the lipids profiles and other metabolism factors.

**Table 1:** The characteristics of the cases and the controls

Groups	*n*	Age (years)	BMI (kg/m^2^)	SBP (mmHg)	DBP (mmHg)	Cr (mmol/l)	BUN (mmol/l)	Uric acid (μmol/l)	GLU (mmol/l)	TG (mmol/l)	TC (mmol/l)	HDL-C (mmol/l)	LDL-C (mmol/l)
Control group	1639	56.9 ± 11.8	24.7 ± 3.6	122.6 ± 13.7	76.6 ± 11.1	71.4 ± 25.3	4.89 ± 1.58	264 ± 79.9	4.75 ± 0.8	1.2 ± 0.9	4.4 ± 0.9	1.29 ± 0.5	2.7 ± 0.8
DM group	1947	57.8 ± 11.9	26.0 ± 3.7	139.5 ± 24.9	85.9 ± 16.4	76.3 ± 32.6	6.30 ± 5.7	287 ± 116	8.1 ± 3.6	3.4 ± 1.8	4.95 ± 2.1	1.16 ± 0.4	2.8 ± 1.9
*t or U*		2.013	10.48	23.75	18.73	3.369	9.621	6.734	37.269	14.913	8.948	−8.98	−1.155
*P*		0.052	<0.001	<0.001	<0.001	0.001	<0.001	<0.001	<0.001	<0.001	<0.001	<0.001	0.041

BMI, body mass index; SBP, systolic blood pressure; DBP, diastolic blood pressure; Cr, creatinine; BUN, blood urea nitrogen; GLU, glucose; HDL-C, high density lipoprotein-cholesterol; LDL-C, low density lipopretein-cholesterol.

### SelS genotype and allele frequencies in the two groups

rs12910524, rs1384565, and rs4965814 were in Hardy–Weinberg equilibrium in the control group (all *P*>0.05, Table 2). However, rs2101171 was not in Hardy–Weinberg equilibrium either in the control group or in the T2DM group (both *P*<0.001). Table 2 shows detailed information for each SNP as well as the allele frequencies. The results show that CC genotype and C allele in rs1384565 are very common in the T2DM group compared with that in the control group (both *P*<0.001). We also found C allele in rs2101171 is more frequent in the T2DM group compared with that in the control group (*P*=0.015). In a multivariable logistic regression model, rs1384565 was found to be an independent risk factor for T2DM. After adjustment of confounding factors such as smoking, alcohol consumption, hypertension, diabetes, as well as serum levels of TG, TC, high-density lipoprotein, the difference remained significant (CC compared with TT, *P*=0.002, OR = 1.884, 95% CI: 1.263–2.811; CT compared with TT, *P*<0.001, OR = 1.764, 95% CI: 1.412–2.204, Table 3).

**Table 2:** Distribution of SNPs in the cases and in the controls

SNPs	Alleles (1/2)	Groups	Genotypes (*n*, %)			P* value	OR* (95% CI)	HWE	MAF	P** value
			1/1	1/2	2/2					
rs12910524	T/C	Case	891 (0.458)	855 (0.439)	201 (0.103)	0.072	1.12 (1.01–1.23)	0.845	0.323	0.022
		Control	696 (0.425)	744 (0.454)	199 (0.121)			0.993	0.348	
rs1384565	C/T	Case	235 (0.121)	902 (0.463)	810 (0.416)	<0.001	1.68 (1.52–1.87)	0.505	0.352	<0.001
		Control	108 (0.066)	583 (0.356)	948 (0.578)			0.154	0.244	
rs2101171	C/T	Case	461 (0.237)	530 (0.272)	956 (0.491)	0.126	0.88 (0.80–0.97)	<0.001	0.373	0.015
		Control	430 (0.262)	454 (0.277)	755 (0.461)			<0.001	0.401	
rs4965814	C/T	Case	336 (0.173)	874 (0.449)	737 (0.379)	0.371	0.974 (0.88–1.07)	0.005	0.397	0.589
		Control	274 (0.167)	774 (0.472)	591 (0.361)			0.446	0.403	

HWE, Hardy–Weiberg equilibrium

*represents the the comparasion of genotypes; **represents the the comparasion of alleles.

**Table 3:** Logistic regression analysis results

	B	S.E.M.	Wald	df	*P*-value	OR	95% CI	
							Lower	Upper
rs1384565s			28.173	2	<0.001			
rs1384565s(1)	0.633	0.204	9.637	1	0.002	1.884	1.263	2.811
rs1384565s(2)	0.568	0.114	24.979	1	<0.001	1.764	1.412	2.204
BMI	0.060	0.016	14.342	1	<0.001	1.062	0.981	1.003
SBP	0.056	0.004	158.006	1	<0.001	1.057	2.678	3.262
DBP	-0.008	0.006	2.106	1	0.147	0.992	1.056	1.322
GLU	1.084	0.050	463.444	1	0.000	2.956	0.823	1.180
TG	0.167	0.057	8.427	1	0.004	1.182	0.492	0.877
TC	-0.014	0.092	0.025	1	0.875	0.986	0.767	1.134
HDL	-0.420	0.148	8.100	1	0.004	0.657	0.492	0.877
LDL	-0.070	0.100	0.487	1	0.485	0.933	0.767	1.134

BMI, body mass index; SBP, systolic blood pressure; DBP, diastolic blood pressure; GLU, glucose; HDL-C, high density lipoprotein-cholesterol; LDL-C, low density lipopretein-cholesterol

## Discussion

In the present study, we found that rs1384565 variations in the *SelS* gene are associated with T2DM in a Chinese population. This was the first study to investigate common allelic variants in *SelS* gene and its association with T2DM in Chinese population.

The human *SelS* gene is located at 15q26.3 [22]. In previous studies, this region has not been identified in genome-wide linkage scans for diabetes-related phenotypes in human population. However, many studies suggested genetic variations in this region were associated with blood glucose levels [12], lipids’ profiles, and obesity [17,21]. Therefore, the *SelS* gene is considered as a candidate gene of T2DM. Also, SelS is a function receptor of SAA, an inflammatory factor, which is reported to be associated with plasma glucose level [23]. Previously, we found SAA1 gene polymorphisms were associated with IMT [24], HDL-C [25], ankle-brachial index (ABI) [26], and plasma uric acid levels [27] which were related to CVD in Chinese population.

In the present study, we performed a case–control study to observe the relationship between genetic polymorphisms of SelS and T2DM. We found rs1384565 CC genotype is very common in the T2DM patients than that in the control subjects. After adjustment in some confounders, the association remains significant, which indicated that rs1384565 CC genotype was an independent risk factor for T2DM.

The mechanisms which may link SelS genetic polymorphism to T2MD are largely unknown. SelS is a 21-kDa transmembrane protein with an extensive histological distribution [12]. Studies have shown that SelS is closely associated with inflammation, oxidative stress, and endoplasmic stress. It acts as a receptor for the acute phase inflammatory response protein (SAA), and an NF-κB binding site is located within the *SelS* gene promoter region [12,28]. Moreover, SelS can regulate the production of inflammatory factors such as IL-1β and IL-6 [29]. Karlsson et al. [30] found that the *SelS* mRNA in the subcutaneous adipose tissues of T2DM patients was significantly increased after hyperinsulinemic–euglycemic clamp experiments. In contrast, no significant change in expression was detected in the healthy control group. Subsequently, another group analyzed *SelS* mRNA expression in omental adipose tissues from T2DM patients and non-T2DM individuals and showed that SelS expression in these tissues was higher in T2DM patients than that in non-T2DM individuals and was positively correlated with the insulin resistance index [31]. The above studies indicated that membrane SelS was closely associated with the glucose metabolic process in the body. SelS expression in the liver, adipose tissue, and skeletal muscle promoted the pathogenesis and development of T2DM and insulin resistance, whereas overexpression of SelS in pancreatic islets protected pancreatic islet β cells from oxidative stress-induced injury.

In conclusion, our study suggested the genetic polymorphisms of *SelS* gene were associated with T2DM. However, the mechanisms which may link SelS genetic polymorphism to T2DM remains unclear.

## Abbreviations

CI: confidence interval
CVD: cardiovascular diseases
FPG: fasting plasma glucose
HDL-C: high desity lipoprotein cholesterol
LDL-C: low density lipoprotein cholesterol
OR: odds ratio
NF-κB: nuclear transcription factor-κB
NGT: normal glucose tolerance
SAA: serum amyloid A
TC: total cholesterol
T2DM: Type 2 diabetes
TG: triglyceride
2hPG: 2-h postload plasma glucose
